# ESR and X-ray Structure Investigations on the Binding and Mechanism of Inhibition of the Native State of Myeloperoxidase with Low Molecular Weight Fragments

**DOI:** 10.1007/s00723-015-0698-8

**Published:** 2015-05-19

**Authors:** Balagopalakrishna Chavali, Thierry Masquelin, Mark J. Nilges, David E. Timm, Stephanie L. Stout, William F. Matter, Najia Jin, Prabhakar K. Jadhav, Gary G. Deng

**Affiliations:** Division of Tailored Therapeutics and Imaging, Lilly Corporate Center, Eli Lilly and Company, Bldg.87/C04, Column S17 DC 1940, 893 S Delaware Street, Indianapolis, IN 46285 USA; Discovery Chemistry Research and Technologies, Lilly Corporate Center, Eli Lilly and Company, 893 S Delaware Street, Indianapolis, IN 46285 USA; Division of Endocrine and Cardiovascular Research, Lilly Corporate Center, Eli Lilly and Company, 893 S Delaware Street, Indianapolis, IN 46285 USA; School of Molecular and Cellular Biology and Illinois EPR Research Center, Illinois EPR Research Center, 506 S. Mathews St., Urbana, IL 61801 USA

## Abstract

As an early visitor to the injured loci, neutrophil-derived human Myeloperoxidase (hMPO) offers an attractive protein target to modulate the inflammation of the host tissue through suitable inhibitors. We describe a novel methodology of using low temperature ESR spectroscopy (6 K) and *FAST*™ technology to screen a diverse series of small molecules that inhibit the peroxidase function through reversible binding to the native state of MPO. Our initial efforts to profile molecules on the inhibition of MPO-initiated nitration of the Apo-A1 peptide (AEYHAKATEHL) assay showed several potent (with sub-micro molar IC50s) but spurious inhibitors that either do not bind to the heme pocket in the enzyme or retain high (>50 %) anti oxidant potential. Such molecules when taken forward for X-ray did not yield inhibitor-bound co-crystals. We then used ESR to confirm direct binding to the native state enzyme, by measuring the binding-induced shift in the electronic parameter *g* to rank order the molecules. Molecules with a higher rank order—those with *g*-shift *R*_relative_ ≥15—yielded well-formed protein-bound crystals (*n* = 33 structures). The co-crystal structure with the LSN217331 inhibitor reveals that the chlorophenyl group projects away from the heme along the edges of the Phe366 and Phe407 side chain phenyl rings thereby sterically restricting the access to the heme by the substrates like H_2_O_2_. Both ESR and antioxidant screens were used to derive the mechanism of action (reversibility, competitive substrate inhibition, and percent antioxidant potential). In conclusion, our results point to a viable path forward to target the native state of MPO to tame local inflammation.

## Introduction

Inflammation arising from the innate defenses is a pervasive clinical parameter of concern across atherosclerosis, diabetes, cancer, chronic kidney disease, and neuro degeneration [1–5]. Our interest is focused on identifying and deriving the mechanism of action (MOA) of inhibitors directed against an inflammatory target protein derived from the innate defense cells.

Neutrophils dominate the innate defense response of the host system and, in response to infection, perform the following actions: extravasation, phagocytosis, degranulation of the microbicidal agents into the lysosomes, and respiratory burst of oxyradicals and their byproducts like HOCl (a powerful antimicrobial detoxifier) [3–6]. In addition, they also employ neutrophil extracellular traps (NETs) that contain proteolytic and DNAses enzymes, dual acting anti-siderophore and cationic protein, and decondensed histones [7]. A central player in all these defense processes (with the exception of phagocytosis) is Myeloperoxidase (MPO), which uniquely catalyzes the reaction between the chloride and hydrogen peroxide to form hypochlorous acid (HOCl). MPO’s role in balancing and regulating the above response toward detoxification without tissue damage is still unraveling [8–10]. MPO is implicated in atherosclerosis through its presence in advanced lesions, oxidation of High Density Lipoproteins (HDL) (via Apo A1), its function as NO oxidase leading to endothelial dysfunction, and by its catalytic release of the metalloproteinases [4, 11]. MPO is a 150 KD protein, having a ferric heme as a catalytic site covalently linked to the protein. As a catalytic pro-oxidant enzyme, MPO presents itself as a viable upstream therapeutic target to manage the cascade of events leading to inflammation.

Current drug discovery approaches to control MPO’s toxic response include [4, 12]

1. Inhibition of NADPH oxidase, a local source of H_2_O_2_ in the neutrophil granules, that fuels MPO’s catalytic action;
2. Scavenging MPO’s oxidant products like HOCl;
3. Inhibition of the catalytic active Compound I with reversible inhibitors;
4. Guiding the catalytic cycle to accumulate the less potent catalytic intermediate, Compound II; and
5. Using suicidal substrates to inactivate the enzyme.

We took an approach that focused on the inhibitor reversibly binding to the heme pocket in the native state of the enzyme (Fe^3+^) as against the catalytic forms Compound I/II in hMPO. In this model, we rationalized that the inhibitor-bound-MPO will hinder peroxide access to the heme (a requirement for the catalytic state generation) [13] and hence may render it dysfunctional both in its intracellular (granular or lysosomal states) and in the extracellular (NETs or plasma bound) forms. Potent nitration of proteins is one among the several microbicidal pathways used by MPO (via Compound I, Approach 3 above) for detoxifying the tissue. We initially used this route to investigate the inhibition of MPO by a library of compounds. However, the results from this approach were ambiguous as it was difficult to delineate the MOA of inhibition from the antioxidant/redox process. We reasoned that the inhibitors, in the midst of an oxidant pool of Ferric (Fe^3+^) and Compound I (Fe(IV)=O), H_2_O_2_, and superoxide/singlet oxygen are either acting as one electron donors (antioxidant pathway) or converted to a co-substrate for the enzyme. Further, many of the potent molecules from this screening method do not co-crystallize with the protein. To obtain direct proof of binding and to steer clear of antioxidant-mediated-inhibition, we employed ESR, to detect direct binding of the inhibitors to the paramagnetic iron in the heme pocket. While our work is under progress, another group reported the use of modified hydroxamates as highly potent (IC50 = 5 nM) and specific reversible inhibitors of the native hMPO [13]. Using Surface plasmon resonance studies, they measured the strength of binding and correlated it with the degree of inhibition of the enzyme. Our studies differ from the above in two aspects. We used a novel methodology (low temperature ESR) to screen molecules for their binding strengths. ESR was complimented with *FAST*™ technology (a method of screening mixtures of small molecule fragments for binding to the protein molecules in the crystalline state) to initiate a fragment based drug design (FBDD) approach for the identification and confirmation of actives [14, 15]. Both ESR and antioxidant screens were then used to derive the mechanism of action (reversibility, competitive substrate inhibition, and percent antioxidant potential). Secondly, we identified non-substrate type of inhibitor scaffolds as inhibitors of the native hMPO.

The current study describes our successful efforts toward identifying molecular scaffolds that bind to the active site in the native state (confirmed with ESR and X-ray results), which do not act via the anti oxidant pathway, and are mechanistically competitive and reversible in binding. We used a diverse set of molecular scaffolds for probing the binding to the MPO active site. These compounds evolved from a SAR design of five structural scaffolds (Manuscript describing the SAR design, syntheses, X-ray structures, a MPO-specific chemiluminescence method to derive the enzyme-inhibition-constants, mouse inflammation model development, and in vivo results is under preparation).

## Experimental Section

### Reagents

For the alpha screen assay, hMPO was obtained from Biodesign (Cat # A31804H, 1.5 mg/ml). For ESR, hMPO was obtained from Athens Research and Technology in a lyophilized from in 50 mM Sodium acetate, pH 6.0 with 100 mM NaCl at a concentration of 1 mg/0.321 ul/vial. For the X-ray work, hMPO was purchased from Lee Biosciences (Catalog # 426-10, St Louis, MO). AlphaScreen^®^ protein A acceptor beads and Streptavidin-coated donor beads were obtained from PerkinElmer (IgG detection kit (Protein A) Cat # 6760617M). Biotinylated peptide substrate (AEYHAKATEHL) was obtained from SynthAssist. Rabbit anti-nitrotyrosine antibody (cat# A21285) was obtained from Molecular Probes. Superblock buffer was from Pierce (Cat# 37535). FBS was obtained from Gibco (Cat 10091-148). 2, 2′-azinobis (3-ethylbenzothiazoline-6-sulfonic acid) (ABTS, Cat# A3219- 100 ml, concentration 1.8 mM), reagent grade H_2_O_2_ (30 % by volume, Cat # H1009), and Potassium Persulfate (Cat # 379824-5G) were obtained from Sigma-Aldrich. Alpha screens and ABTS decolorization assays employed sterile 96-well plates (Corning, Cat # 3688 and #3603), respectively.

### Inhibitors

A large library of compounds (*n* ~ 2500) were tested for binding to MPO to inhibit its catalytic activity. All the compounds reported in this paper were obtained from various commercial sources. Proprietary compounds are not included in this paper. Inhibitors are dissolved in 100 % DMSO for the stock solution and were appropriately diluted with 100 % DMSO or an appropriate buffer as required in the assay. A total of *n* = 350 compounds were tested as inhibitors against the native state (Fe^3+^) of MPO by ESR.

### Crystallography

Human Myeloperoxidase and co-crystals of MPO were crystallized and analyzed using methods similar to those described by Davey and Fenna [16, 17]. Briefly, hanging drops of 25 mg/mL MPO, 25 mM sodium acetate pH 5.0, 50 mM NaCl, 50 mM ammonium sulfate, 2 mM calcium chloride, and 3-10 % polyethylene glycol 8000 were set up at 21 °C over reservoir solutions containing 300 mM sodium chloride. Crystals of the space group P21 and having two MPO subunits per asymmetric unit grew with the aid of micro-seeding as very thin plates having a brownish orange tinge. Crystals were soaked with inhibitor compounds at a concentration of 50 mM, in a stabilizing buffer containing 18 % polyethylene glycol 8000, 56 mM ammonium sulfate, 2.2 mM calcium acetate, and 56 mM HEPES pH 7.0. Co-crystals were cryo-protected in a stabilizing buffer containing 22.2 % methylpentanediol prior to flash cooling in liquid nitrogen.

Diffraction data were collected at 100 K using Synchrotron beam line 31ID at the Advanced Photon Source (Argonne National Laboratory) and were integrated using MOSFLM v.6.2.6 [18]. The structure was solved (Table 1 shows the details) by rigid body refinement against the diffraction data using a starting model derived from Protein Data Bank entry 1D7W [19]. Inhibitor binding to the MPO heme group was indicated by appropriately shaped peaks in the Fo–Fc electron density maps. The inhibitor was included in the model, and iterative cycles of manual model building with COOT [20] and restrained refinement with REFMAC v5.2.0019 [21] were used to complete the overall structure. Figures of the crystal structure were generated using PYMOL (www.pymol.org). Mean B-factors for the inhibitor molecules bound in each of the A- and B-subunit active sites are 27.4 and 36.1 Å^2^, respectively, which are significantly higher than the B-factors of 8.6 and 11.0 Å^2^ associated with the respective A- and B-subunit heme groups. The higher B-factors for the inhibitor molecules could be attributed to inhibitor occupancies of less than 1.0 or to higher degrees of thermal motion associated with the inhibitors. Lower than expected electron density peak heights were also observed for the Cl atom in the inhibitors, which could be due to the radiation damage to this X-Ray sensitive group.

**Table 1:** Data collection (values in parenthesis are for the highest resolution shell of 1.71–1.62 Å)

Space Group, P21 Cell dimensions, 92.2 Å × 63.5 Å × 111.2 Å, *β* (beta) = 97.3º Resolution, 1.62 Å Rsym, 0.142 (0.814) Mean *I*/*σI*, 4.9 (1.6) Completeness (%), 100 (100) Redundancy, 3.7 (3.6) Refinement (values in parenthesis are for the highest resolution shell of 1.662–1.620 Å) Resolution, 1.62 Å No. Reflections, 160977 (11830) Rwork/Rfree, 0.178/0.201 R.M.S deviations Bond lengths, 0.01 Å Bond angles, 1.3º	

### Alpha Screen Assay

In a typical experiment, MPO (11.8 nM final concentration) in 20 mM phosphate buffer pH 7.4, 10 % glycerol and 0.005 % gelatin (PB) is added to each well of a multi-well plate containing 20 μl PB ± compound (final concentration of DMSO = 0.9 %). The reaction is started by the addition of 20 μl PB containing 1.5 μM ApoAI peptide substrate, 300 μM diethylenetriaminepentaacetic acid (DTPA), 300 μM sodium nitrite, and 30 μM H_2_O_2_. The total reaction volume is 60 μl. The plates are incubated with agitation for 45 min at RT. The reaction is terminated with the addition of 20 μl of a stop solution containing 11.2 nM rabbit anti-nitrotyrosine antibody (final conc. of 2.8 nM), 100 μg/ml streptavidin donor beads, and 100 μg/ml protein A acceptor beads (final concentration of 40 μg/ml), FBS (1.6 % final concentration) in Superblock buffer. The plates are sealed, incubated in the dark for 1 h, and read on a Packard Fusion alpha plate reader.

### ESR Binding Assay

Sodium acetate (0.1 M, with and without 100 mM NaCl according to the assay requirement) pH 6 buffer containing 25 % n-propanol was used as the reaction buffer for ESR. In a typical binding assay, the following are sequentially added in an eppendorf tube: 50 μl of 20.8 μM MPO (4.6 μM final concentration) and 15 μl of inhibitors in DMSO at 40× concentrations to the enzyme followed by 170 μl of the buffer. After 5-min. incubation at room temperature, the entire reaction mixture (0.235 mL) was transferred to the ESR quartz tubes. To avoid the quartz tubes breaking during the rapid freezing cycles, the ESR tubes were initially frozen in a 1:1 Isopentane-Cyclohexane mixture cooled with dry ice powder and then transferred to a liquid nitrogen Dewar to be queued up for ESR measurements. The reaction mixture contained a final concentration of 6 % DMSO. ESR spectra were recorded on a Varian E-12, X-band spectrometer equipped with an Air Products liquid helium cryostat. The other instrument settings are as follows: sample temperature, 5 K; *n* = 7 scans; $\upsilon$ = 9.0482 GHz; microwave power, 10 dB; 579 A/Ds per point; 1.00 min. scan; time constant 0.032 s; gain:3.20E + 3; and Modulation amplitude 10.00 G. Magnetic fields were calibrated with an NMR Gaussmeter.

### Reversible Binding Assay

We performed the following two assays for independently confirming the reversibility of binding. In the filtration method, hMPO stock (1.5 mg/ml) is diluted to 5 μg/ml with 20 mM phosphate buffer pH 7.4, 10 % glycerol (no gelatin). 120 μl of this solution is mixed with an equal volume of the compounds at a concentration of 30 μM (final concentration 10 μM), followed by mixing in the plate and incubation for 15 min. at room temperature. 120 μl aliquot of this solution is transferred into separate YM30 Micron filter units and centrifuged 6 min at 9000 rpm, followed by the addition of 300 μl of the buffer and further centrifugation for 12 min. at 9000 rpm. Wash and centrifuge cycles are repeated 2 more times with 300 μl buffer, each followed by 12 min. centrifugation steps. The protein left on the membrane is mixed with the PB buffer to a final volume of 120 μl and subjected to Alpha screen method as described before to probe the nitration of Apo A1 peptide substrate which is taken as a confirmation of the restoration of the catalytic activity of the protein.

In the ESR protocol, the methodology of Hori et al. [22] was applied with some modifications.The protocol followed is similar to the binding assay except that a higher concentration of hMPO (9.2 μM final) is used. This is necessary as multiple washings followed by dilutions lead to considerable loss of the enzyme due to its retention on the membrane. After 5 min of incubation in the eppendorf at room temperature, the contents were transferred to an Amicon Ultra-4 tube (5 ml capacity) and filled with 4 ml of 0.1 M sodium acetate buffer (containing 50 mM NaCl) pH 6.0. Using spin conditions of 4000×*g* in a swinging bucket rotor at 20 °C, the samples are washed repeatedly (2×) followed with a final wash containing 1 ml of the sodium acetate buffer (chloride free) + 25 % n-propanol and then concentrated. The concentrated solution ca. 300 μl is transferred to ESR quartz tubes and measured at 4 K. A higher concentration of MPO in these reversible binding assays enabled a good ESR signal over noise from the washed (3X) sample.

### ABTS Radical Cation Decolorization Assay to Measure the Antioxidant Capacity

The assay was modeled after the published procedure [23]. Briefly, ABTS radical cation was prepared by adding 100 μl of 70 mM K_2_S_2_O_8_ to 10 ml ABTS, followed by vortexing and incubating at 37 °C water bath for 2 h. The solution turns blue with a strong absorbance at 734 nm (blank absorption). The MPO inhibitors were dissolved in DMSO, and further dilutions were made in water (dilution plate: Falcon Cat. # 351172). Trolox standards are dissolved in water. The reaction is started by adding the ABTS radical cation solution to the MPO inhibitors or Trolox (final concentration 20 μM). The plate is gently shaken for 1 min and then set on a bench top at room temperature for 30 min. The plate is then read at 734 nm on a Spectromax M5. The data are analyzed as % inhibition at 20 uM single dose treatments.

### Peroxidase Inhibition Assay

Hydrogen peroxide (30 %, v/v; 9.71 M) is used with different dilutions in ice-cold deionized water. MPO at 50 μl of 20.8 μM (4.6 μM final concentration) is incubated with different concentrations of H_2_O_2_ in the absence and in the presence of inhibitors (inhibitor to MPO concentration is 40:1) for 1 min and rapidly frozen at 77 K. The inhibitors are added to MPO, incubated for 5 min prior to H_2_O_2_ addition in these reactions.

## Results and Discussion

### Inhibition of the MPO-Initiated Apo-A1 Nitration Pathway

Through a chloride independent pathway, MPO catalyzes the one electron oxidation of nitrite ions to nitrogen dioxide radicals which then nitrosylate protein amino acids like Tyrosine [24]. We used the Apo-A1 peptide substrate (AEYHAKATEHL) to follow the nitration of the Tyrosine (indicated by Y in the scheme below):

$$\begin{aligned} {\text{MPO }} + {\text{ H}}_{ 2} {\text{O}}_{ 2} \to {\text{ Compound I}} \hfill \\ {\text{NO}}_{ 2}^{ - } + {\text{ Compound I}} \to {\text{ NO}}_{ 2}^{ \cdot } + {\text{ Compound II}} \hfill \\ \end{aligned}$$

**Fig. 1:**
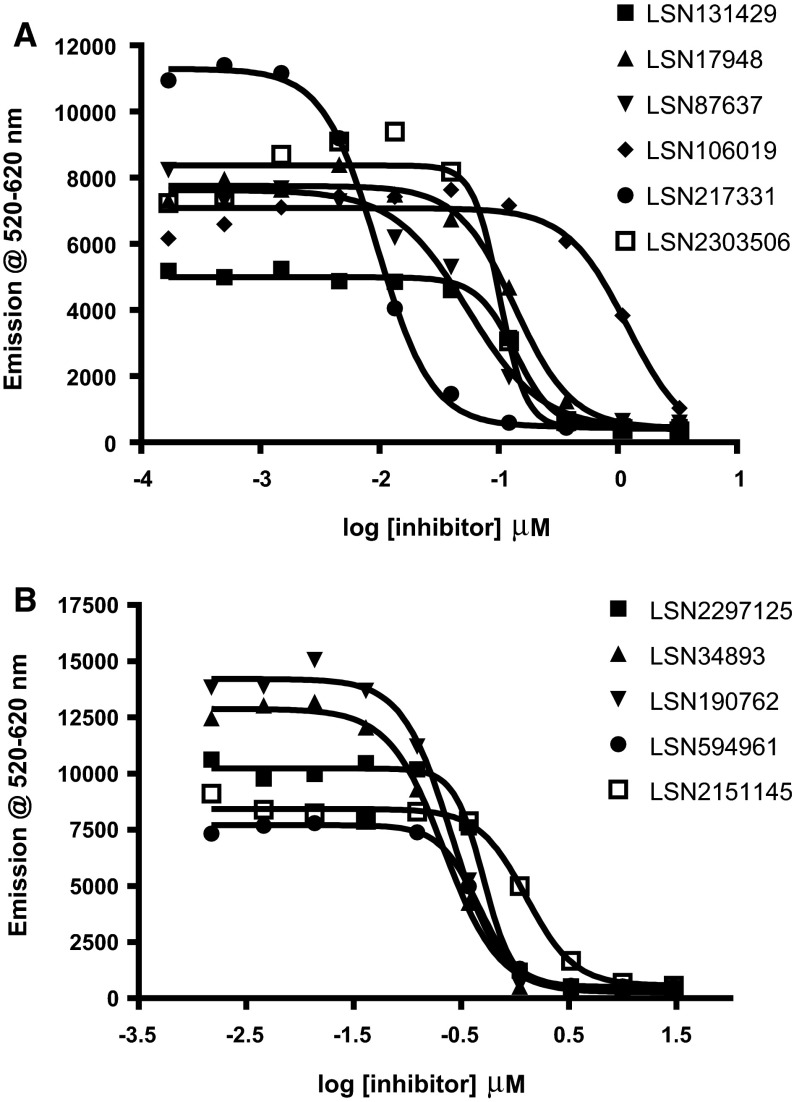
Concentration response curves from the alpha screen assay run on a representative set of the compound library profiled for inhibition of the MPO’s catalytic nitration of Apo-A1 nitration. **a** Compounds that bind to the heme (as found later with ESR), **b** non-binding compounds. The IC50 values for these compounds are collected in Table 2

Our initial experiments focused on identifying inhibitors that bind to the heme to inhibit the above catalytic action. A representative set of inhibitors is collected in Fig. 1a (strong binders, vide infra) and b (non binders, vide infra). The IC50s for these compounds are indicated in Table 2. As shown in Table 2, several potent inhibitors, with desirable IC50 (≤10 μM), were found by this procedure.

**Table 2:**
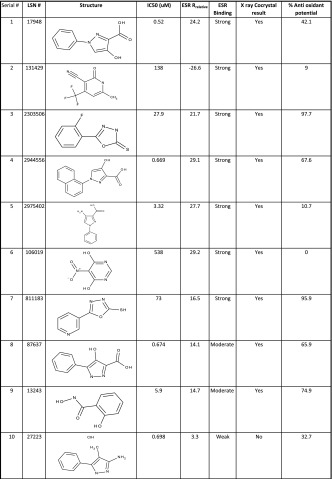

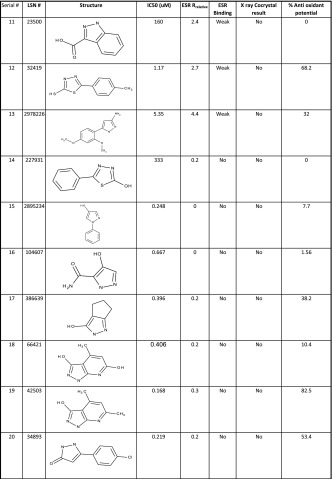

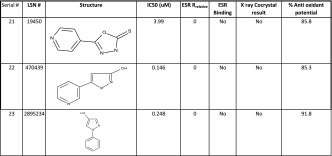
A small subset of hMPO inhibitors used to describe the mechanism of action (MOA) in terms of their binding (IC50, μM), rank ordering from ESR, success of co-crystal growth from X-ray and percent anti oxidant potential

Previous studies [13, 25–28] have shown that compounds inhibit the above pathway by either coordinating with the heme iron or by scavenging the radicals (antioxidant) or acting as substrate to Compound I. The latter two are not a desired route for the native state inhibition. But the predominant contribution in the above nitrosylation inhibitory mechanism is not apparent from the observed IC50 values in Table 2. In our studies, we focussed on avoiding the antioxidant-mediated-inhibition by using an ABTS assay [23] to filter compounds based on a threshold of 50 % above which the compounds are classified as strong anti oxidants. Those compounds that showed the desirable profile of IC50 ≤10 μM and antioxidant capacity <50 % (Table 2 shows only a representative set) were taken forward for the crystallization of inhibitor-bound MPO crystals. However, we had very little success in isolating bound X-ray quality co-crystals from these compounds. Evidently, these spurious molecules (a subset collected separately in Fig. 1b) are acting as competitive electron donors to Compound I or II or in some cases acting as substrates. This necessitated an alternate method to unambiguously confirm the inhibitor binding to the protein.

### ESR Spectra is Sensitive to Inhibitor Binding

We employed ESR, a technique that uniquely describes the putative inhibitors binding to the heme iron in MPO. Binding induces electronic changes in the heme active site symmetry which manifests itself in shifting the *g* values [22] and thus can be used as a sensitive probe of the protein conformation. The tetrapyrrole containing heme unit is usually planar (D_4h_) in the absence of interactions with the protein. When the heme gets attached to the apo protein, the heme plane is distorted. These distortions are brought about by the protein through covalent binding to heme [29, 30]. The geometry of the water molecules in the heme pocket also plays a role. These distortions modulate the redox properties of the heme iron. Further distortions (and hence further lowering of the symmetry) are expected to happen when the inhibitor molecules enter the heme pocket to coordinate with the heme or with the water molecules. ESR is a sensitive technique to track the effect of these structural distortions on the d-orbital symmetry of the iron. Hence, ESR at liquid helium temperatures was used to assess inhibitors binding to the heme. In addition, in some of the bound complexes, the snap freezing of the reaction mixture containing the inhibitor and enzyme enables us to capture both the bound and unbound states (on/off states) in the solution state (vide infra).

**Fig. 2:**
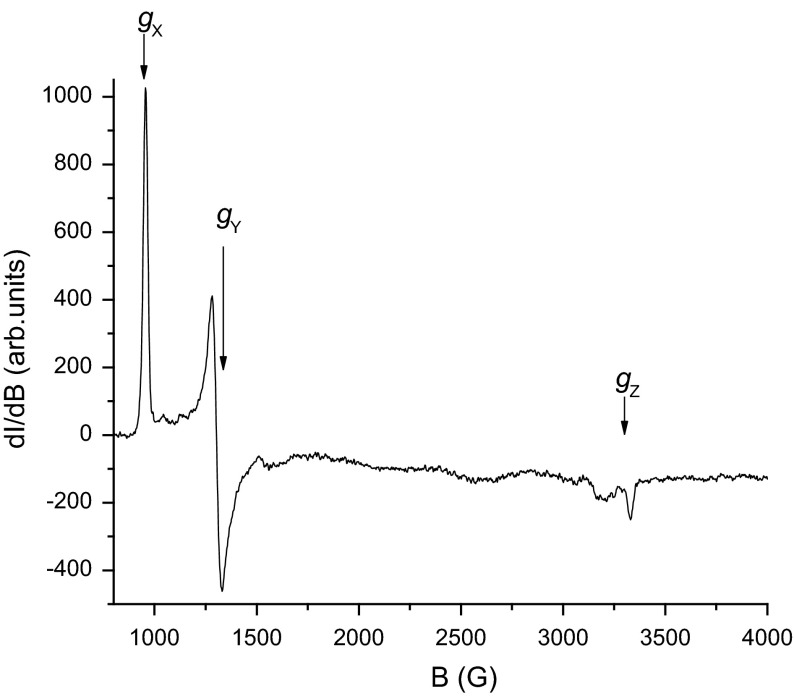
X-band ESR spectrum of hMPO showing the high-field and low-field signals (800–4000 G). *g*_*X*_, *g*_*Y*_ and *g*_*Z*_ represent the rhombic components of the electronic *g* tensor. The instrument settings are given in the experimental section

ESR spectra of the native state of the hMPO enzyme in pH 6 buffer is rhombic at 4 K and showed a predominantly high-spin Fe^3+^ spectrum with *g*_*X*_ = 6.778 *g*_*Y*_ = 4.978, and *g*_*Z*_ = 2.00 (Fig. 2).

A signal at *g* ~ 4.3 (at ~1550 G Fig. 1) from high-spin rhombic Fe^3+^ represents a small amount of non-heme iron; it is invariably present because the middle Kramers’ doublet (±3/2) is almost magnetically isotropic and has high transition probability. The broad signal on the low-field side of *g*_*Z*_ ~ 2 in Fig. 1 is due to a cavity contaminant. The ESR spectrum of the native state (Fig. 1) is most probably a representative of the resting enzyme in the phagosome where the pH is low and the chloride concentration is high [31]. Masao-Ikeda et al. [31] studies have revealed that axial water coordination increases rhombicity along with a mixture of low- and high-spin Fe^3+^ signals. Our enzyme preparations contained 0.05 M NaCl in sodium acetate buffer at pH 6. At these conditions, to the best of our knowledge, there is little prior evidence of water or hydroxyl coordination to the 6th axial position of the heme.

This spectral behavior is reflective of Fe in a distorted heme plane bonded to a protein with a proximal histidine and a weak (distal) axial coordination by chloride [31]. The experimental *g* values correspond to the spin Hamiltonian pertinent to the high-spin ion (*S* = 5/2) [29].

1 $$H = D\left( {S_{Z}^{2} - 1/3 \, S\left( {S + 1} \right) \, + E/D\left( {S_{X}^{2} {-} \, S_{Y}^{2} } \right)} \right) + \, \beta g_{e} S.B_{e}$$

where *D* and *E* are the eigen values of the axial and rhombic field components, i.e., the symmetry of the ligand (inhibitors approaching heme in this case). In axially symmetric systems (tetragonal symmetry with *E/D* = 0), the ESR behavior predominantly reveals a two line spectrum with *g*_*X*_ = *g*_*Y*_ = 6, and *g*_*Z*_ = 2. When the heme lodges inside the protein, the protein side chain amino acid (histidine) approaches the heme vicinity and either binds (axially) directly to the iron in the heme pocket. This results in the deviation from the axial symmetric environment. Thus, the fifth ligand leads to lower (rhombic) symmetries causing the signal at *g* ~ 6 to split. This non-tetragonal splitting of the heme is quantitated by the relationship [29],

2 $$E/D = \, \Delta g/ 4 8$$

where ∆*g* represents the absolute difference between the *g*_*X*_ and *g*_*Y*_ components. Alternatively, making use of the fact that E/D maximum value = 1/3, one can simplify the above expression as a percentage of rhombicity, *R*:

3 $$R \, = \, \left( {\Delta g/16} \right) \, \times \, 100 \, \%.$$

The native heme in MPO itself has an axial symmetry with small rhombic distortion, reflected by *g*_*X*_ ≠ *g*_*Y*_ ≠ *g*_*Z*_. To rank-order molecules in terms of their ability to distort the heme pocket/plane environment, we used a ratio of total distortion in the bound complex w.r.t. distortion seen in the native unbound enzyme:

4 $$R_{\text{relative}} = \, \left( {\left( {R_{\text{bound}} {-} \, R_{\text{native}} } \right) \, \text{ / }R_{\text{native}} } \right) \, \times \, 100.$$

The *g*_*Z*_ region (~2, Fig. 2 field range 3340–3550) was not used due to the fact that *g*_*Z*_ is fairly insensitive to *E*/*D*, when *E*/*D* is small (i.e., nearly axial). From the relative rhombicities of the bound and unbound complex, a measure of the strength of binding could be derived. We have used this *R*_relative_ (Eq. 4) as a sensitive measure of the distortion of the native heme environment brought about by the incoming inhibitor molecule. For the ease of classification, we have empirically defined those with 5 ≤ *R*_relative_ ≥ 2 as weak binding; 15 ≥ *R*_relative_ ≥ 10 as moderate binding; and 25 ≥ *R*_relative_ ≥ 15 as strong binding (Table 2). This classification was used to select only the moderate and strongly bound molecules for testing the co crystal growth for the X-ray crystal structure (vide infra).

No other iso forms of MPO are observed under these conditions. No low-spin signals are seen. Previous studies on the bovine MPO identified both the high- and low-spin signals at 77 K [22]. However, the authors did not report the concentrations of the enzyme used in those ESR studies. We could not reproduce this ESR behavior in our batches of hMPO obtained from the vendor. The ESR signal intensity of the hMPO (at a concentration 23.07 uM) decreases, as the temperature is raised from 5 to 77 K. This decrease at a higher temperature (77 K relative to 5 K), however, is not accompanied by the appearance of low-spin signals, i.e., no evidence of temperature dependence in the spin state equilibrium could be detected. We henceforth used 4–7 K for all our binding studies in ESR.

**Fig. 3:**
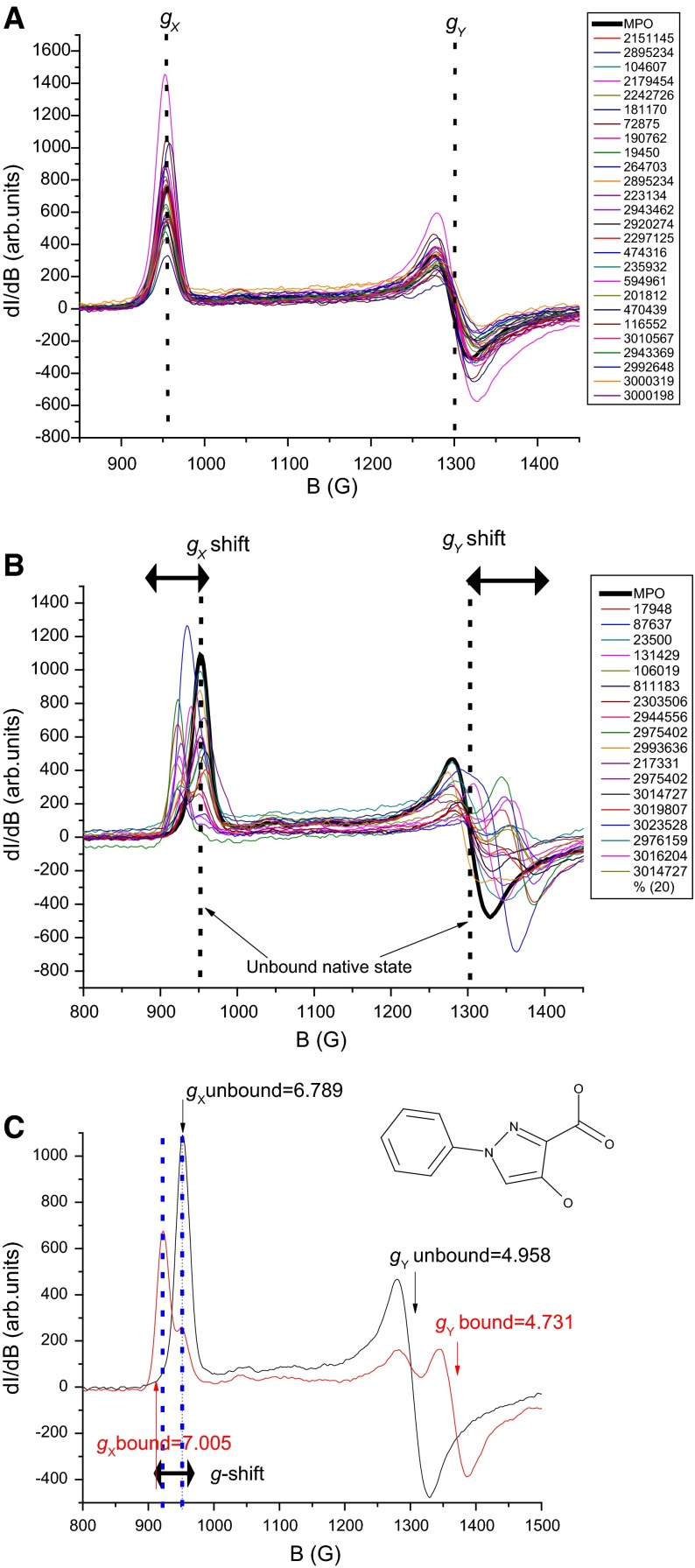
X-band ESR spectra in the low-field region (800–1450 G) is a sensitive measure of the heme plane distortions on binding. **a** Indicates absence of binding of the structurally different scaffolds of compounds to the heme iron as indicated by the non-variance of the *g*_*X*_ and *g*_*Y*_ signals. The native hMPO signal is shown in *bold black* as a visual aid to depict the non shifting of *g* values of the native enzyme with the addition of purported inhibitors. **b** Significant shifts in the ESR resonances on binding in the *g*_*X*_ and *g*_*Y*_ regions. A majority of the inhibitors shift the native hMPO signal in the positive side (difference between bound versus unbound *g* values is positive, Δ*g* > 0) due to different distortions on the heme plane symmetry. **c** LSN17948 binding accompanied by greater/positive ∆*g*, i.e., 30 % of the enzyme is unbound while 70 % is bound. The reaction volume in the ESR tube is 235 μl. MPO 50 μl (23.07 μM final), 15 μl inhibitor in 100 % DMSO, Inhibitor:MPO ratio is 40:1. DMSO final concentration is 6 %. buffer 170 μl (0.1 M Sodium acetate containing 100 mM NaCl pH 6 + 25 % n-propanol)

We were justified in using ESR to confirm binding when we found that many of the compounds which were considered potent inhibitors (IC50 ≤10 μM) from the alpha screen assay (Table 2; Fig. 1b) did not affect the ESR spectra of native hMPO as can be seen in the Fig. 3a. In contrast, when the putative inhibitors bind to MPO, the ESR signals at *g*_*X*_ and *g*_*Y*_ (800–1450 G) were broadened or even split into two components, signifying the formation of bound complexes (Fig. 3b). In the spectra of the some of the bound-complexes, there is still a significant proportion of the unbound enzyme. This enables one to calculate the percent or proportion of the bound versus the unbound enzyme complex (Fig. 3c). The *g* values of the bound complexes are collected in Table 3.

**Table 3:** Electronic *g* values of a representative set of the bound complexes

S. No.	LSN	*g*_*X*_	*g*_*Y*_	Δ*g*
1	17,948	6.995	4.739	2.256
2	87,637	6.917	4.827	2.09
3	23,500	6.789	4.914	1.875
4	131,429	6.559	5.215	1.344
5	106,019	7.023	4.713	2.31
6	811,183	6.924	4.809	2.115
7	2,303,506	6.9749	4.799	2.1759
8	2,944,556	7.0681	4.7601	2.308
9	2,975,402	7.006	4.735	2.271
10	2,993,636	6.976	4.793	2.183
11	217,331	6.901	4.819	2.082
12	3,019,807	6.948	4.773	2.175
13	3,023,528	7.036	4.725	2.311
14	2,976,159	7.007	4.705	2.302
15	3,016,204	7.023	4.725	2.298
16	3,014,727	7.007	4.739	2.268

The *g*_Z_ is ~2.00 for all the complexes. Δ*g* is the absolute difference between *g*_*X*_ and *g*_*Y*_

Due to low S/N ratio in the high-field region (weak signal on a high cavity background at 5 K), *g*_*Z*_ value is not accurately determined

For ESR, we used inhibitor at 40× concentration to the enzyme. In some cases when this concentration is lowered to 4×, the proportion of the bound versus unbound changed. This could be, in part, due to the fact that since the inhibitor-protein interactions are largely non-covalent and weak, some of the displaced water molecules in the heme environment now return to restore the symmetry and thereby affect the bound:unbound ratios. Alternatively, since DMSO (used for dissolving the inhibitor used at 6 % final concentration) also competes for the binding with the heme, the inhibitor-induced distortion (and hence the bound:unbound ratio) is changed. Since we have used ESR mainly in binary mode (to detect the presence or absence of binding) only, these dilution-induced changes in the proportion of the on/off states did not change our end points.

## Solution State ESR Results are Confirmed with X-ray Crystal Structures

To test whether the solution state results hold well in the solid state and further to decrease the incidence of unbound crystals, we then attempted to co-crystallize the enzyme with the moderate (15 ≥ *R*_relative_ ≥ 10) and strong binding (25 ≥ *R*_relative_ ≥ 15) series of compounds. In all the cases, we obtained high-quality co-crystals (*n* = 33). In each of these cases, the compounds were located inside the heme pocket and are found to bind either directly to the heme iron or to the side chains via the water molecules.

**Fig. 4:**
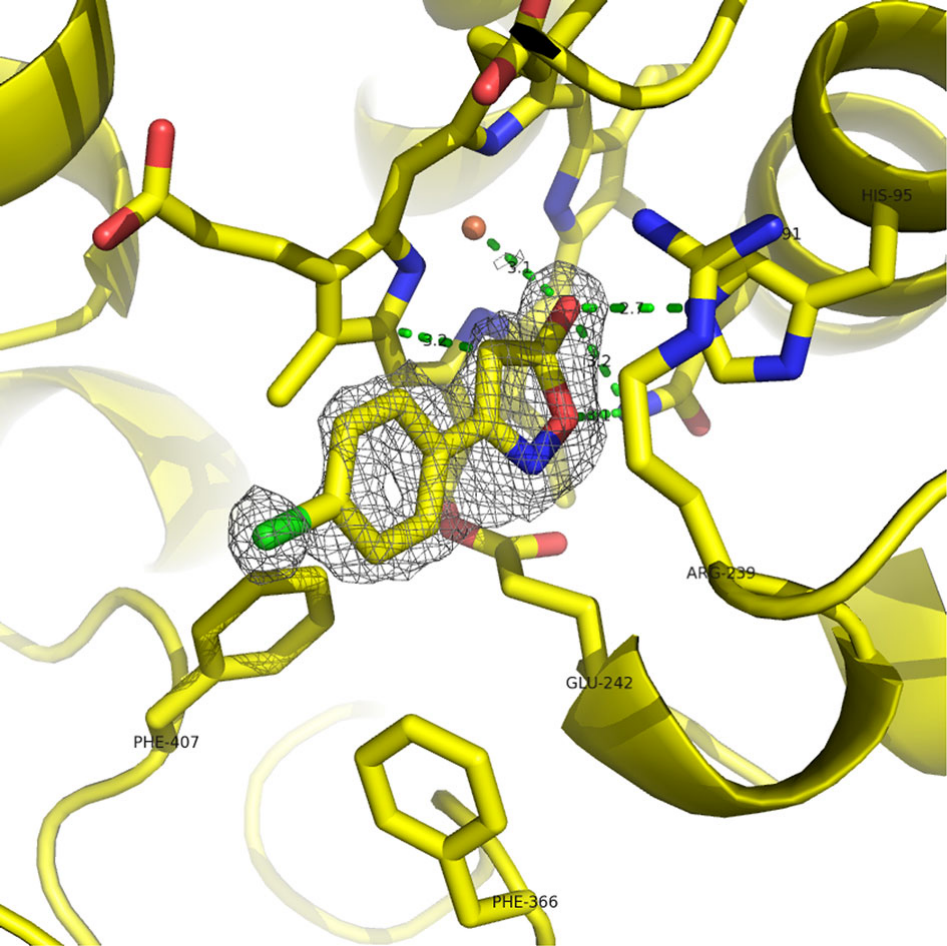
Binding mode of the inhibitor LSN217331 to the MPO active site. Carbon, oxygen, nitrogen, chlorine, and iron atoms are colored *yellow*, *red*, *blue*, *green* and *orange*, respectively. Close intermolecular contacts are indicated by *dashed green lines* with distances labeled in Å. The protein backbone is displayed as a *yellow ribbon* diagram, with side chains of key residues modeled as sticks. Fo–Fc electron density map, contoured at 3 *σ* is displayed as *gray mesh*

The crystal structure of 217331 bound to MPO (Fig. 4a, b) reveals a large number of specific interactions between the inhibitor and enzyme, thus providing a clear structural rationale for the inhibitory mechanism. The compound hydroxyl group makes close direct contacts to the heme iron ion (3.1 Å), the Gln91 side chain amide nitrogen (3.2 Å) and the His95 imidazole NE2 (2.7 Å). These non-bonded contact distances are consistent with the hydrogen bonds to the side chains and a favorable dipole interaction with the iron ion. The isoxazole O atom also contacts the Gln91 side chain amide nitrogen (3.1 Å), while the ring *N* atom makes contacts of 3.1 and 3.4 Å with the two methylene carbon atoms from the Glu242 side chain. One side of the isoxazole ring contacts the gamma and delta methylene C atoms of Arg239 in a nearly parallel orientation, while the other side lies against the heme prosthetic group at an angle of approximately 50º, with the CH contacting the porphyrin at 3.2 Å. The chlorophenyl group projects away from the heme along the edges of the Phe366 and Phe407 side chain phenyl rings. Occupation of this space by the inhibitor would sterically block peroxide and co-substrates (e.g., chloride) from reacting at the heme center of the active site. Indeed, the inhibitor sterically blocks both the peroxide binding site between the iron ion and His95 and the halide binding site near the Gln91 side chain amide [16].

### MOA

#### Reversible Binding

**Fig. 5:**
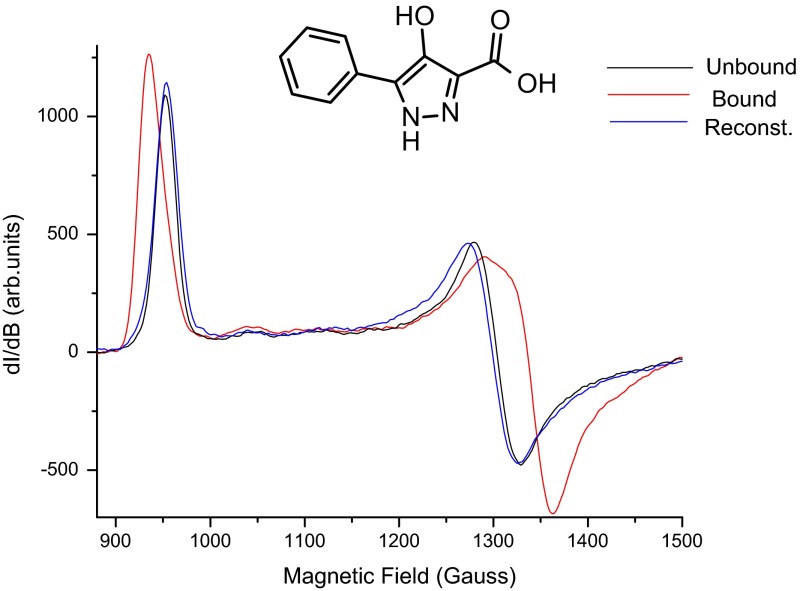
X-band ESR spectra (800–1450 G) depicting the reversible nature of the inhibitor binding to hMPO. Only a representative example (LSN87637) is shown. After incubation of the compound with hMPO for 5 min, the bound complex was washed with buffer ± chloride on an Amicon desalting tube followed by concentration to 250 μl for ESR measurements

Since the *g*_*X*_ and *g*_*Y*_ regions are very sensitive to the binding of compounds in the heme pocket, we next assessed the reversibility of the binding of these compounds. We used two methods (Filtration and ESR) to independently verify the reversibility of the binding. In every case of the strong and moderately strong binders tested, the shifted ESR spectra of the bound complexes return to the native unbound state after washing. Figure 5 shows a representative example from a moderately strong binder (LSN87637) series.

#### Substrate (H_2_O_2_) Inhibition

We used ESR to gain additional insights on the competitive nature of the compounds to inhibit peroxidase action, a key requirement for our native state binders. Since ESR and X-ray have clearly established the binding of the compounds in the vicinity of the heme, we focussed on the mode of action of these compounds (with moderate and strong *R*_relative_) to classify the MOA.

Using heme-H_2_O_2_ systems, several studies have tracked and characterized the protein and hydroperoxyl radicals with concomitant formation of ferryl (Fe=O)^3+^ species [32, 33]. In our studies, we focused only at the high-spin region (*g* ~ 6) ignoring the *g* ~ 2 region where free radicals dominates [33].

**Fig. 6:**
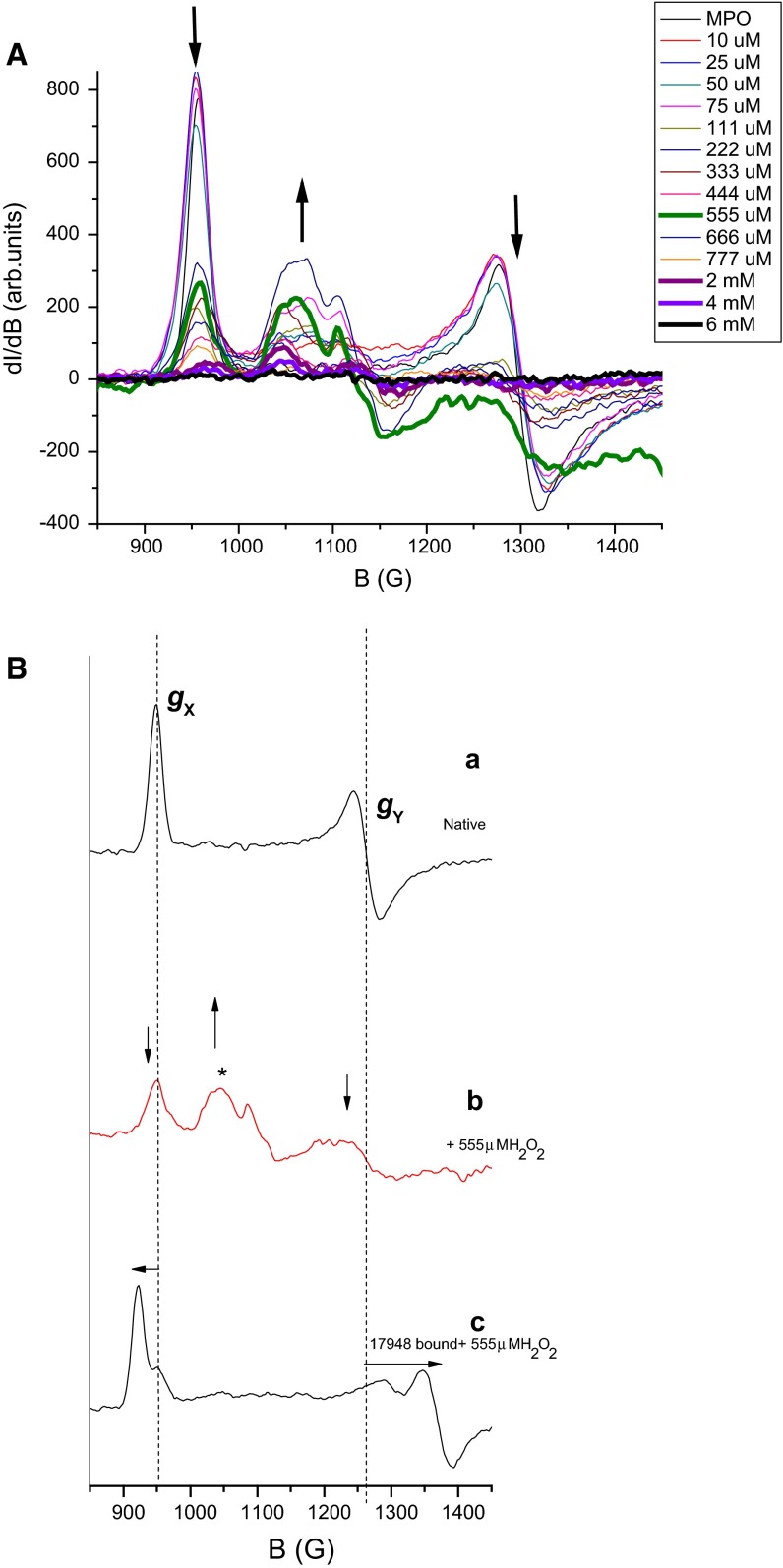
**A** X-band ESR spectra (800–1450 G) of hMPO exposed to different concentrations of H_2_O_2_. As the H_2_O_2_ concentration (*label inset)* increases, the *g*_*X*_ and *g*_*Y*_ signals (*arrows* indicate the direction of change) decrease with a corresponding increase in *g*_intermediate_ = 6, i.e., the rhombic pattern (*g*_*X*_, *g*_*Y*_, and *g*_*Z*_) gradually changes into *g*_intermediate_ (axial). **B** Inhibitor LSN 17948 (15 μl in 100 % DMSO) is added to hMPO (50 μl) before H_2_O_2_ (555 μM) is introduced in the reaction mixture. The spectrum is unchanged and no *g*_intermediate_ signal is seen. In **B**, *a* and *b* are taken from **A** and shows decay (*down arrows*) of *g*_*X*_ and *g*_*Y*_ in the presence of 555 μM H_2_O_2_, the sign * indicates the growth of signal corresponding to denaturation, while *c* shows the absence of peroxide-induced decay in the presence of 17948 (the binding-induced *g*-shifts are indicated with *horizontal arrows*). The reaction volume in the ESR tube is 235 μl. hMPO 50 μl (23.07 μM final), 15 μl of H_2_O_2_ at the indicated concentrations, buffer 170 μl (0.1 M Sodium acetate containing 100 mM NaCl pH 6 + 25 % n-propanol)

Figure 6A shows the spectral changes in the low-field region of the native enzyme as H_2_O_2_ is varied. As the peroxide concentration increases, the *g*_*X*_ and *g*_*Y*_ signals drop down in intensity with a concomitant increase in the *g* ~ 6 region (*B* value ~ 1050 G). A similar behavior is reported in the presence of denaturing agent like guanidine HCl [34]. A likely explanation for these effects is the snapping of the covalent (via the axial histidine) and non-covalent (hydrogen bonds and other van der Wall contacts) interaction-driven control by the protein on the heme conformation resulting in the tetrapyrrole unit returning to its near planar (higher symmetry) state. This is a dynamic reversible process with a threshold, above which, the heme with its restored planarity, is ejected from the enzyme [35]. The ESR spectrum of MPO in the presence of H_2_O_2_ indicates that the rhombicity (*g*_*X*_ ≠ *g*_*Y*_ ≠ *g*_*Z*_) is gradually replaced with more axial symmetry (*g*_*X*_ = *g*_*Y*_, and *g*_*Z*_).

We then added the inhibitors (with 25 ≥ *R*_relative_ ≥ 10) prior to the introduction of H_2_O_2_ and followed the ESR changes in this region. In the presence of the bound inhibitors, these changes are prevented (Fig. 6c) even at very high concentrations of H_2_O_2_ (555 μM, hMPO: H_2_O_2_ 1:20 Fig. 6b), and the spectrum is identical to the native human MPO (albeit shifted due to inhibitor binding). The native *g*_*X*_ ~ 6.778 signal does not go down in intensity. In contrast, Forbes et al. [13] showed that the MPO-bound hydroxamates are metabolized by MPO in the presence of H_2_O_2_. The presence of bound inhibitors inside the heme pocket blocks the entry of H_2_O_2_ (as seen in the co-crystal structures above) and prevents the denaturation of the enzyme. It has been reported [4] that H_2_O_2_ >50 μM causes enzyme denaturation and the new ESR signal at *g* = 6 (indicated by ‘*’ in Fig. 6b) is a probable fingerprint of that process. The absence of these spectral changes in the presence of bound inhibitors clearly indicates that the access of H_2_O_2_ into the heme pocket either at the gate or to the 6th axial coordination site (distal side) is prevented by these inhibitors. We found that DMSO can also mimic this process of restricting peroxide access and coordination to the heme. In order to differentiate the inhibitor role from DMSO, we used hydroxamic acid inhibitors which are water soluble and found these to bind to the enzyme and prevent the peroxide-induced changes on the enzyme even in the absence of DMSO.

In conclusion, the results represent a comprehensive analysis of the electronic structural changes of MPO induced by compound libraries of different scaffolds. Using low temperature ESR, our studies provided several lead molecules (for e.g., LSN 17948, LSN294456, LSN87637, LSN2975402, etc.) with the following traits:

- Inhibitor of the MPO enzyme in its catalytic state;
- Reversible in its action;
- Well defined binding mode as shown by inhibitor-bound X-ray crystal structures;
- MOA dominated by inhibitor binding to the active site and preventing the access of the heme to incoming H_2_O_2_ rather than acting as an anti oxidant;
